# Case-control study of the *PERIOD3* clock gene length polymorphism and colorectal adenoma formation

**DOI:** 10.3892/or.2014.3667

**Published:** 2014-12-11

**Authors:** MELANNIE ALEXANDER, JAMES B. BURCH, SUSAN E. STECK, CHIN-FU CHEN, THOMAS G. HURLEY, PHILIP CAVICCHIA, MEREDITH RAY, NITIN SHIVAPPA, JACLYN GUESS, HONGMEI ZHANG, SHAWN D. YOUNGSTEDT, KIM E. CREEK, STEPHEN LLOYD, XIAOMING YANG, JAMES R. HÉBERT

**Affiliations:** 1 South Carolina Statewide Cancer Prevention and Control Program, University of South Carolina, Columbia, SC, USA; 2 Department of Epidemiology and Biostatistics, Arnold School of Public Health, University of South Carolina, Columbia, SC, USA; 3 Dorn Department of Veterans Affairs Medical Center, Columbia, SC, USA; 4 Center for Molecular Studies, Greenwood Genetic Center, Greenwood, SC, USA; 5 Bureau of Epidemiology, Division of Disease Control and Health Protection, Florida Department of Health, Tallahassee, FL, USA; 6 Division of Epidemiology, Biostatistics and Environmental Health, School of Public Health, University of Memphis, Memphis, TN, USA; 7 College of Nursing and Health Innovation, and College of Health Solutions, Arizona State University, Phoenix, AZ, USA; 8 Department of Drug Discovery and Biomedical Sciences, South Carolina College of Pharmacy, University of South Carolina, Columbia, SC, USA; 9 South Carolina Medical Endoscopy Center, and Department of Family Medicine, University of South Carolina School of Medicine, Columbia, SC, USA; 10 Medical Chronobiology Laboratory, Dorn Department of Veterans Affairs Medical Center, Columbia, SC, USA; 11 Department of Family and Preventive Medicine, School of Medicine, University of South Carolina, Columbia, SC, USA

**Keywords:** adenoma, clock gene, circadian rhythm, colorectal cancer, variable number tandem repeat

## Abstract

Clock genes are expressed in a self-perpetuating, circadian pattern in virtually every tissue including the human gastrointestinal tract. They coordinate cellular processes critical for tumor development, including cell proliferation, DNA damage response and apoptosis. Circadian rhythm disturbances have been associated with an increased risk for colon cancer and other cancers. This mechanism has not been elucidated, yet may involve dysregulation of the ‘period’ (*PER*) clock genes, which have tumor suppressor properties. A variable number tandem repeat (VNTR) in the *PERIOD3* (*PER3*) gene has been associated with sleep disorders, differences in diurnal hormone secretion, and increased premenopausal breast cancer risk. Susceptibility related to *PER3* has not been examined in conjunction with adenomatous polyps. This exploratory case-control study was the first to test the hypothesis that the 5-repeat *PER3* VNTR sequence is associated with increased odds of adenoma formation. Information on demographics, medical history, occupation and lifestyle was collected prior to colonoscopy. Cases (n=49) were individuals with at least one histopathologically confirmed adenoma. Controls (n=97) included patients with normal findings or hyperplastic polyps not requiring enhanced surveillance. Unconditional multiple logistic regression was used to calculate odds ratios (ORs) with 95% confidence intervals (CIs), after adjusting for potential confounding. Adenomas were detected in 34% of participants. Cases were more likely to possess the 5-repeat *PER3* genotype relative to controls (4/5 OR, 2.1; 95% CI, 0.9–4.8; 5/5 OR, 5.1; 95% CI, 1.4–18.1; 4/5+5/5 OR, 2.5; 95% CI, 1.7–5.4). Examination of the Oncomine microarray database indicated lower *PERIOD* gene expression in adenomas relative to adjacent normal tissue. Results suggest a need for follow-up in a larger sample.

## Introduction

According to recent estimates, over 136,000 new patients and more than 50,000 deaths occurred in 2014 in the USA due to colorectal cancer (CRC), which makes it the third most common and deadly cancer among both men and women (1). Colorectal adenomatous polyps are the primary precursor lesions for CRC, accounting for 85–90% of cases (2). Developing a better understanding of factors related to adenoma susceptibility and progression thus represents an important goal for CRC prevention.

Disruption of circadian rhythms or clock gene expression is emerging as a novel and potentially modifiable cancer risk factor, although the pathophysiological mechanism is incompletely understood (3,4). The central circadian pacemaker is located in the suprachiasmatic nuclei (SCN) of the anterior hypothalamus. Generation of circadian rhythms is accomplished primarily via photic input from the retina, which synchronizes the reciprocal transcriptional-translational expression of at least nine core clock genes: *PER1*, *PER2*, *PER3*, *CRY1*, *CRY2*, *CLOCK*, *BMAL1*, *TIM* and *CK1ɛ* (3–5). In most tissues, this system facilitates the diurnal expression of ~10% of the entire mammalian genome via genetic and epigenetic regulation of clock-controlled genes (6–10). Various factors, such as shift work, late bedtimes or poorly timed light exposure can disrupt endogenous circadian timing, thus altering clock gene expression and the cellular processes they help regulate (4). Since clock genes regulate processes that are considered hallmarks of carcinogenesis (cell cycle control, DNA damage response, apoptosis), their dysregulation may serve as an underlying biological mechanism linking altered circadian rhythms with cancer (4,11–15). Clock gene polymorphisms have been associated with non-Hodgkin’s lymphoma and cancers of the breast and prostate (4). Clock gene polymorphic variation also influences sleep regulation (5,16,17), which may contribute to increases in cancer susceptibility that have been observed among people who experience circadian rhythm disturbances or sleep disruption (18–21). For example, shift work and sleep disturbances have been associated with increased CRC risk (20,22–24) and truncated sleep (<6 h/night) has been associated with an increased odds of colorectal adenoma formation relative to adenoma-free controls (18).

The *period* (*PER*) clock genes have immunomodulatory (5,25,26) and tumor suppressor properties (4,11,19,27,28). Mutation or altered expression of *PER* genes has been observed among cancer patients relative to controls, within human tumors relative to adjacent normal tissue and in experimental cancer bioassays (4,11,27,29–36). Whether differential expression of *PER* or other clock genes occurs in human adenomas versus normal tissue is not known. The *PER3* variable number tandem repeat (VNTR; rs57875989) length polymorphism contains 4 or 5 copies of a 54-bp sequence encoding 18 amino acids. The 5-repeat variant adds several potential phosphorylation motifs to the gene, and *PER3′*s interaction with circadian processes may be enhanced among those individuals (16,37). The 5-repeat *PER3* allele is associated with a relatively penetrant phenotype that includes morning circadian preference (16,38,39), increased cognitive decline in response to sleep deprivation (16), differences in levels or timing of melatonin or cortisol secretion (37,40,41), and a tendency towards depressive symptoms or an earlier onset of bipolar disorder (42,43). *PER3* is considered a candidate tumor suppressor gene (27,28,33), and the 5/5 *PER3* VNTR genotype has been associated with increased premenopausal breast cancer risk (33), though not consistently (21,44). Recently, the relationship between the *PER3* VNTR and CRC risk was examined in Greece and no association was observed, although a relatively small portion of the study population was homozygous for the 5-repeat allele (<2%), and differences in genotype frequency among cases and controls were not adjusted for potential confounding (45). The role of *PER3* or other clock genes in human adenoma formation has yet to be examined in detail. Therefore, this exploratory study tested the hypothesis that adenoma cases are hetero- or homozygous for the 5-repeat *PER3* variant relative to adenoma-free controls.

## Materials and methods

Participants and data from two different endoscopy centers in the Columbia, SC metropolitan area were pooled for this analysis; the South Carolina Medical Endoscopy Center (Site 1, n=93), and the WJB Dorn Veterans Administration Medical Center (DVAMC; Site 2, n=53). Eligible patients were English literate adults 30–80 years who were scheduled for a screening or surveillance colonoscopy at either site. The present study was approved by the Institutional Review Boards (IRB) of the DVAMC and the University of South Carolina prior to informed consent and enrollment. Participants provided a peripheral blood sample for DNA recovery and completed a questionnaire to ascertain information on: demographic (gender, marital status, income, race, ethnicity), lifestyle (smoking history, diet, physical activity), and occupational (employment status, job industry, type of shift, history of shift work) factors, as well as personal and family history of cancer and other chronic diseases. Individuals who were getting a colonoscopy due to symptoms (presence of gastrointestinal bleeding, hematochezia, melena, fecal occult blood, iron deficiency or constipation) were collapsed into the screening category due to low counts (n=10). Cases were defined as individuals with at least one histologically confirmed adenoma, and controls were subjects with a normal colonoscopy or a normal biopsy not requiring heightened surveillance (e.g., hyperplastic polyp).

Genomic DNA was extracted and genotyping for the *PER3* VNTR sequence was performed using previously described methods (42,46). For participants recruited from Site 1, the *PER3* VNTR sequence was amplified via polymerase chain reaction (PCR) using the following forward (5′-TGGCAGTGA GAGCAGTCCT-3′) and reverse (5′-AGTGGCAGTAGGATGG GATG-3′) primers (33,44). The final 20 μl PCR reaction mixture was made up of 1 μl (20 ng) of genomic DNA, 10 μl of OneTaq^®^ Hot Start 2X Master Mix with standard buffer (20 mM Tris-HCl, 22 mM KCl, 22 mM NH4Cl, 1.8 mM MgCl2, 5% glycerol, 0.06% Igepal CA-630, 0.05% Tween-20, 0.2 mM dNTPs, 25 U/ml OneTaq Hot Start DNA Polymerase; New England BioLabs, Inc., Ipswich, MA, USA), 1 μl (0.375 μM) of each oligonucleotide primer and 7 μl of PCR-grade water. The reactions were heated to 94°C for 2 min followed by 35 cycles at 94°C for 30 sec, 60°C for 30 sec and 72°C for 45 sec. Finally, the reactions were extended for 7 min at 72°C using the S1000 Thermal Cycler (Bio-Rad, Hercules, CA, USA). PCR products were then separated by electrophoresis on a 3% agarose gel. For Site 2, after DNA extraction, 200 ng of genomic DNA was subjected to PCR. The PCR primers used for Site 2 assays were: 5′-CAAAATTTTATGA CACTACCAGAATGGCTGAC-3′ (forward) and 5′-AACCTT GTACTTCCACATCAGTGCCTGG-3′ (reverse). The resulting reaction mixture consisted of 25 μl standard PCR buffer, 5% DMSO, 1.0 mM MgC1_2_, 0.2 mM dNTP, 1 U Taq polymerase (Gibco-Invitrogen, Carlsbad, CA, USA), and 0.4 μM of each oligonucleotide primer. PCR cycling conditions were as follows: 3 min at 94°C; 35 cycles of 30 sec at 94°C, 30 sec at 58°C and 30 sec at 72°C; and at 72°C for 30 sec, PCR products were extended using a Perkin-Elmer GeneAmp System 9700 (Waltham, MA, USA). A 2% agarose gel stained with ethidium bromide was used to separate and visualize the PCR fragments at 220 V for 30 min. Both primers provide valid characterization of the *PER3* VNTR (33,42,44–48). DNA sequences of amplicons produced by each set of primers were verified via Sanger sequencing. Duplicate genotyping was performed in 10% of all samples from both sites for quality control purposes, and there was 100% concordance among duplicates (42). Hardy-Weinberg equilibrium (HWE) was examined, and gene frequencies for the *PER3* VNTR were in HWE among the entire study population (p=0.74), and among controls from both sites (p=0.99) or within each site (Site 1, p=0.94; Site 2, p=0.82, data not shown).

Statistical analyses were performed using the Statistical Analysis Software (SAS^®^) computer program (version 9.2; SAS Institute, Cary, NC, USA) and the R meta-analysis package (version 2.14.1; http://cran.r-project.org). Potential differences between study variables by case status within the entire study population, and within each site separately, were examined using the Chi-squared test for differences between proportions. Unconditional multiple logistic regression was used to estimate odds ratios (ORs) with 95% confidence intervals (95% CIs) for *PER3* VNTR genotype among adenoma cases relative to controls using the 4/4 genotype as referent (33,42). Co-variates considered for inclusion in adjusted models were known or suspected adenoma or CRC risk factors [age, gender, race, body mass index (BMI), CRC family history, smoking history, work-related factors, diet, vitamin and supplement use, physical activity, sociodemographic characteristics (income, education), personal medical history], recruitment site and variables that differed among participants and non-participants (gender, personal cancer history, ulcer diagnosis, lactose intolerance, secondhand smoke exposure). Final models included variables that were statistically significant (p≤0.05) in the saturated model, or produced at least a 10% change in the parameter estimate for genotype (decision latitude at work, procedure reason, recruitment site). Differences in median shift work duration by *PER3* genotype were compared using the Wilcoxon rank sum test. Ancillary analyses examined relationships between lifetime shift work and adenoma status; or *PER3* genotype and adenoma status after stratification by procedure reason (screening vs. surveillance colonoscopy), or shift work (none vs. any, or median split on years of lifetime shift work). Random-effects meta-analysis was used to evaluate the study hypothesis since recruitment occurred at two separate sites. The I^2^ and Q statistics were used to assess heterogeneity between sites. Finally, Oncomine (49), a publically accessible gene expression microarray database, was queried to examine potential differential expression of *PER1*, *PER2* and *PER3* clock genes in adenomas versus adjacent normal tissue.

## Results

The median age of the participants was 58 years (25–75th percentile: 53–64 years), and they were primarily European American (EA) (65%), and male (72%). Most (69%) participants had engaged in at least one year of shift work (median, 3 years; 25–75th percentile: 0–12.3 years). Adenomas were detected in 34% of participants (females, 28%; males; 36%). Compared to controls, cases were more likely to have smoked (78 vs. 56%), have higher workplace decision latitude (57 vs. 29%), and have undergone a surveillance rather than a screening colonoscopy (59 vs. 40%, Table I). The following characteristics were different by site, regardless of case status (p≤0.05, data not shown): gender, race, marital status, income level, ever smoking, age group, reason for procedure and number of years of lifetime shift work. Compared to controls, cases in Site 1 were less likely to be diagnosed with diabetes, more likely to have smoked in the past and more likely to undergo a surveillance colonoscopy (data not shown). Site 2 cases were older compared to controls (data not shown).

Overall *PER3* VNTR genotype frequencies (4/4, 47%; 4/5, 42%; 5/5, 11%) were consistent with those reported previously (21,33,37–48,50). Adenoma cases were more likely than controls to possess one or two copies of the 5-repeat sequence (4/5 OR, 2.1; 95% CI, 0.9–4.8; 5/5 OR, 5.1; 95% CI, 1.4–18.1; 4/5+5/5 OR, 2.5; 95% CI, 1.7–5.4, Table II). The distribution of lifetime shift work history varied by *PER3* VNTR genotype (Table III). Those with the 4/4 genotype had greater median lifetime years of shift work (5 years) compared to those with the 4/5 (2 years, p=0.02), 5/5 (0.75 years, p=0.05) or the combined genotype (4/5+5/5, 1 year, p<0.01). Shift work or the combined effect of shift work and genotype was not related to adenoma case status (data not shown). When analyses were stratified by procedure reason, the 5/5 genotype was associated with adenoma status among screening patients (OR, 10.7; 95% CI, 1.4–80.7), although CIs were wide and no statistically significant relationship was observed among those with other combinations of genotype and procedure reason (data not shown). When the data were evaluated using meta-analytic methods, adenoma cases were ~2–3 times more likely than controls to have at least one 5-repeat allele, although the CIs were wide and did not achieve statistical significance (4/5 OR, 2.27; 95% CI, 0.43–11.62; p_heterogeneity_, 0.12; I^2^, 60%; 5/5 OR, 3.02; 95% CI, 0.72–12.71; p_heterogeneity_, 0.84; I^2^, 0%; 4/5+5/5 OR, 2.35; 95% CI, 0.60–9.21; p_heterogeneity_, 0.14; I^2^, 54%, data not shown).

Data for *PER1*, *PER2* and *PER3* expression in adenomas relative to normal tissue were retrieved from the Oncomine microarray database (Table IV) (51–53). A statistically significant reduction in *PER3* expression was observed in adenomas relative to normal tissue among each of the available data sets; similar differences were noted for *PER1*, and to a lesser extent *PER2* expression (Table IV).

## Discussion

Few studies have examined the role of the *PER3* VNTR on cancer-related outcomes (21,33,44,45). To our knowledge, this exploratory study is the first to examine the relationship between the *PER3* VNTR and human adenoma risk. Adenoma cases were ~2–5 times more likely to possess the 5-repeat *PER3* length polymorphism compared to controls. Quality criteria for genotyping and colonoscopy were satisfactory (54,55), and adjustment for potential confounding by known or suspected adenoma risk factors did not alter the interpretation of the results. The meta-analysis indicated that the strength of association between *PER3* genotype and adenoma status was generally consistent with the main analysis and the results were not strongly impacted by heterogeneity between the sites. Some imprecise risk estimates with wide confidence intervals were observed due to a limited sample size, particularly for the stratified analyses. Thus, examination of possible effect modification by factors such as race, chronotype or procedure indication (screening vs. surveillance) would benefit from a larger sample in future studies. Nonetheless, the lower bound of the confidence intervals suggest an increased risk for adenoma formation of at least ~40% among homozygous 5-repeat *PER3* variants. *PER* gene expression was not performed among cases and controls in the present study, thus changes in expression relative to the *PER3* VNTR genotype could not be evaluated. However, our query of the Oncomine database indicated that *PER3* and to a lesser extent *PER1* and *PER2* expression was reduced among adenomas compared to normal mucosa, which is consistent with previous studies that observed a reduction in *PER1* and *PER3* expression in human colorectal tumors relative to adjacent normal tissue (32,36,56,57).

The spectrum of known genetic susceptibility markers does not fully account for all CRC cases. For example, 10 loci identified from genome-wide association studies had population attributable risks ranging from 1.7 to 11.9% (58), and another study reported that up to 35% of CRC cases are due to heritable factors (59). The present study mirrors previous investigations that have examined clock gene polymorphisms in conjunction with cancer susceptibility (4,21,33,44,45), including one that identified an association between the 5-repeat *PER3* VNTR sequence and increased odds of premenopausal breast cancer (33). Evidence suggests that *PER3* may function as a tumor suppressor. A recent study among *PER3* knockout mice indicated that 36% of the homozygous null variants developed chemically-induced mammary tumors compared to 12% among heterozygotes and 0% among wild-type mice (27). Another recent study used methylation arrays and stringent selection criteria to screen >14,000 genes to identify putative tumor suppressors associated with human hepatocellular carcinoma; *PER3* was one of only three candidate tumor suppressor genes identified (28). Chronic gastrointestinal inflammation is important for adenoma and CRC development (2), and since *PERIOD* genes play a role in immune system regulation, their expression may influence these processes (5,25,26). Recently, another *PER3* polymorphism (rs2797685) was associated with inflammatory bowel disease, a known CRC risk factor (60). The mechanism whereby *PER3* may exert a tumor suppressor function is currently unknown. The clock genes exert genetic and epigenetic regulatory effects that facilitate the circadian expression of ~5–10% of the entire mammalian transcriptome (6–10), including other known tumor suppressors and oncogenes (e.g., *c-Myc*, *p53*) (31,61,62). Clock genes also help regulate cellular processes that are active during carcinogenesis (cell proliferation, DNA damage response, apoptosis), and clock gene dysregulation may foster adenoma formation by influencing these pathways (4,11,13,15).

Individuals with the 5-repeat *PER3* VNTR sequence tend to have relatively penetrant phenotypic characteristics including delayed sleep phase syndrome, increased susceptibility to cognitive impairment after sleep deprivation, morning circadian preference and differences in the timing or levels of circadian hormone secretion (16,37,40), although some inconsistencies have been reported (21,39,63–65). Whether alterations in sleep and other circadian processes can increase cancer susceptibility remains to be determined, although studies of shift work and cancer incidence suggest this is possible (3,4,24). In the present study, participants with the 5-repeat allele reported less cumulative shift work experience relative to those with the 4/4 genotype (Table III). Additional research is needed to determine whether this is a chance finding or if individuals carrying these variants are less tolerant of shift work and self-select out of these occupations relative to those with the 4/4 genotype. Individuals with the 5-repeat allele may be more susceptible to disturbances in circadian timekeeping (16,37,40,41). For example, those carrying the 5/5 *PER3* variant were sensitive to light-induced melatonin suppression whereas 4/4 homozygotes were not responsive (41). Since melatonin has potent antioxidant, antiproliferative and anti-inflammatory properties in the gastrointestinal tract, a reduction in its secretion (e.g., by exposure to light at night) may facilitate physiologic changes that predispose to increased risks for CRC or other cancers (3,4,20,22,23). Although *PER3* has tumor suppressor properties and its length polymorphism tends to have relatively penetrant phenotypic characteristics, the role of these factors in cancer susceptibility, if any, remains to be characterized.

In conclusion, the present study indicates that individuals with the 5-repeat *PER3* length polymorphism may be more susceptible to adenoma formation. The results are consistent with Oncomine data indicating that *PERIOD* clock gene expression is reduced in adenomas relative to normal GI tissue. Further interrogation of interrelationships between the *PER3* VNTR and genetic or epigenetic pathways that may facilitate adenoma risk, such as changes in the expression of clock-controlled, cancer-related genes, is recommended. Further elucidation of the *PER3* VNTR genotype in relation to circadian rhythm or clock gene dysregulation may lead to development of novel, modifiable targets for adenoma and CRC prevention.

## Figures and Tables

**Table I:** Demographic characteristics of study population.

Variable^a^	Total population (n=146) n (%)	Controls (n=97) n (%)	Cases (n=49) n (%)	Cases vs. controls P-value^b^
Sex				0.32
Male	105 (72)	67 (70)	38 (78)	
Female	40 (28)	39 (30)	11 (22)	
Race				0.12
European American	94 (65)	58 (60)	36 (73)	
African American	51 (35)	38 (40)	13 (27)	
Marital status				0.46
Unmarried	38 (26)	27 (28)	11 (22)	
Married	107 (74)	69 (72)	38 (78)	
Education				0.75
Up to High School	52 (36)	34 (35)	18 (37)	
Some College	40 (28)	25 (26)	15 (31)	
College Undergraduate or Post-Graduate Degree	53 (37)	37 (39)	16 (33)	
Income level				0.37
Under $50,000	63 (46)	40 (45)	23 (49)	
≥$50,000 to $100,000	53 (39)	38 (43)	15 (32)	
>$100,000	20 (15)	11 (12)	9 (19)	
Body mass index (kg/m^2^)				0.22
Normal (≤25)^c^	33 (23)	19 (20)	14 (29)	
Overweight (>25)	113 (77)	78 (80)	35 (71)	
Family history of colorectal cancer				0.84
Yes	25 (17)	17 (18)	8 (16)	
No	120 (83)	79 (82)	41 (84)	
Diagnosis of diabetes				0.15
Yes	42 (29)	24 (25)	18 (38)	
No	102 (81)	71 (75)	31 (62)	
History of smoking				0.01
Ever	91 (63)	53 (56)	38 (78)	
Never	53 (37)	42 (44)	11 (22)	
Work decision latitude^d^				0.03
Often or always	51 (35)	28 (29)	23 (57)	
Never or sometimes	21 (14)	18 (19)	3 (6)	
Unknown	74 (51)	51 (53)	23 (47)	
Age group (years)				0.11
30–54	45 (31)	34 (35)	11 (22)	
55–65	66 (46)	44 (46)	22 (45)	
>65	34 (23)	18 (19)	16 (33)	
Reason for colonoscopy				0.03
Screening	78 (53)	58 (60)	20 (41)	
Surveillance	68 (47)	39 (40)	29 (59)	
Lifetime shift work (years)	Median (25th, 75th percentile)			
	
	3 (0, 12.3)	5 (0, 15)	2 (0, 10)	0.10

a Number of subjects for each variable category may not equal total number of subjects due to missing data;

b Chi-squared test for differences in proportions or Wilcoxon rank sum test for differences in medians between cases and controls;

c n=3 subjects in the underweight BMI category (≤18.5 kg/m^2^);

d Defined by the question: ‘Do you have a good deal of say in decisions about your work?’.

**Table II:** *PER3* VNTR genotype by adenoma status.

Genotype	Controls (n=97) n (%)	Cases (n=49) n (%)	Crude OR	95% CI	P-value	Adjusted OR^a^	95% CI	P-value
4/4	52 (54)	16 (33)	Ref	-	-	Ref	-	-
4/5	38 (39)	24 (49)	2.1	0.9–4.4	0.06	2.1	0.9–4.8	0.07
5/5	7 ( 7)	9 (18)	4.2	1.3–13.0	0.01	5.1	1.4–18.1	0.01
4/5 or 5/5	45 (46)	33 (67)	2.4	1.7–4.9	0.02	2.5	1.7–5.4	0.02

a Adjusted for, decision latitude at work, recruitment site, procedure reason; OR, odds ratio; CI, confidence interval; VNTR, variable number tandem repeat.

**Table III:** Relationship between *PER3* VNTR genotype and lifetime shift work exposure.

		Lifetime shift work (years)			
			
	n (%)	Median	25th Percentile	75th Percentile	P-value^a^
4/4	67 (47)	5	1	15	-
4/5	61 (42)	2	0	10	0.020
5/5	16 (11)	0.75	0	8.5	0.050
4/5 or 5/5	77 (53)	1	0	10	0.008

a Wilcoxon rank sum test for group differences in median duration shift work by genotype using the 4/4 genotype as the referent (n=2 subjects with missing data on lifetime shift work).

VNTR, variable number tandem repeat.

**Table IV:** *PERIOD* gene expression in human adenomas vs. normal tissue^a^.

Referent tissue	Pathological tissue type	*PER1*	P-value	*PER2*	P-value	*PER3*	P-value	Ref.
Normal colon epithelium (n=22)	Colorectal adenoma epithelium (n=56)	−1.3	0.003	−1.2	0.050	N/A	N/A	(51)
Normal colon (n=32)	Colon adenoma (n=25)	−1.7	0.008	−1.2	0.003	−2.3	<0.001	(52)
Normal colon (n=32)	Rectal adenoma (n=7)	−1.9	0.050	1.3	0.880	−1.7	0.001	(52)
Normal colon (n=10)	Colon adenoma (n=5)	−1.0	0.370	1.6	0.990	−2.1	<0.001	(53)
Normal colon epithelium (n=10)	Colorectal adenoma epithelium (n=5)	−1.2	0.050	1.2	0.940	−1.7	0.005	(53)

a Fold-change in mRNA expression in adenomas relative to adjacent normal tissue (number of tissue samples evaluated in parentheses).

Source, www.oncomine.com.
